# Work-related musculoskeletal disorders and associated factors among bank workers in Addis Ababa, Ethiopia: a cross-sectional study

**DOI:** 10.1186/s12199-020-00866-5

**Published:** 2020-07-27

**Authors:** Dereje Dagne, Solomon Mekonnen Abebe, Atalay Getachew

**Affiliations:** 1 Department of Environmental and Occupational Health and Safety, College of Medicine and Health Science, Institute of Public Health, University of Gondar, Gondar, Ethiopia; 2 Department of Human Nutrition, College of Medicine and Health Science, Institute of Public Health, University of Gondar, Gondar, Ethiopia

**Keywords:** Bank workers, Musculoskeletal disorder, Prevalence, Work related

## Abstract

**Background:**

Work-related musculoskeletal disorders (WMSDs) are dramatically increased in the world due to the advancement of technology and competitiveness of markets. There were limited studies carried out regarding WMSDs among bank workers in Africa particularly in Ethiopia. Therefore, the aim of this study was to assess the magnitude of work-related musculoskeletal disorders and associated factors among bank workers in Addis Ababa, Ethiopia.

**Methods:**

Institution-based cross-sectional study design was employed in the study. Multi-stage sampling techniques were used to select 838 bank workers from 62 banks in Addis Ababa. Self-administered standard Nordic questionnaires were used as well. Multivariable binary logistic regression analyses were employed to identify factors associated with WMSDs. Moreover adjusted odds ratio (AOR), 95% confidence interval (CI) and *p* value < 0.05 was used to show the strength of association between explanatory variables and dependent variable.

**Results:**

Out of 838 total numbers of participants, 755 bank workers returned their questionnaires responding with a rate of 90%. Of these, 77.6% (*N* = 586) suffered WMSDs with a 95% CI [75–81%]. Based on the final multivariate logistic regression analysis being female [AOR = 2.98, 95% CI 1.91–4.65], sitting back in a twisted position [AOR = 3.59, 95% CI 2.13–6.08], sitting back bent [AOR = 4.06, 95% CI 2.48–6.66], work on fixed position [AOR = 1.78, 95% CI 1.17–2.71], no work time break [AOR = 3.33, 95% CI 1.44–7.71], type of chairs [AOR = 2.62, 95% CI 1.19–5.75] and job stress [AOR = 2.33, 95% CI 1.19–4.54] were factors significantly associated with WMSDs.

**Conclusion:**

From the study’s findings, the magnitude of work-related musculoskeletal disorders among bank workers was high. Being female, awkward posture, no work time break, fixed position, type of chairs, and job stress are the factors significantly associated with WMSDs. So bank workers should use proper types of chairs, practice proper work posture, increase healthy working conditions, and create awareness programs on how to maintain beneficial health conditions which may lead to increased leisure time.

## Background

Musculoskeletal disorder is a serious problem that every human being will face at least once throughout their lifetime. Work-related musculoskeletal disorder (WMSDs) is a soft-tissue disorder of non-traumatic origin that is caused or exacerbated by interaction with the work environment [1–4]

Every year over 2.34 million women and men die at work from an occupational injury or disease; of these, over 350,000 deaths are due to fatal accidents and almost 2 million deaths are due to fatal work-related diseases [5, 6]. The distribution of work-related fatalities by United Nation (UN) geographical regions accounted Europe for 11.7%, Oceania for 0.6%, Africa for 11.8%, America for 10.9%, and Asia 65.0% [7]. A study conducted in Kuwait and India which focused on 12-month prevalence of WMSDs accounted 80.0 % and 83.5% respectively [8, 9]. In addition, a study conducted in Nigeria and Ghana revealed that a 12-month prevalence of WMSDs among the bank workers accounted for 71.7% and 83.5% respectively [10, 11].

Moreover, the global compensation cost of WMSDs accounted for 40% in 2015. This shows that WMSDs is one of the leading causes of socio-economic burden of workers due to direct and in direct costs [7]. The major factors that may influence the problem includes people sitting with their backs bent, back twisted, having no work time break, psychosocial factors, repetitive work and awkward posture [1].

The studies conducted in different countries showed that office workers including bank workers are vulnerable groups for different WMSDs [8–11]. However, there are limited studies carried out regarding WMSDs among bank workers in Africa particularly in this study setting. Hence, the focus of this study was to assess the magnitude of WMSDs and associated factors among bank workers.

## Methods

### Study design and study population

Institution-based cross-sectional study design was employed. This study was conducted in Addis Ababa the capital city of Ethiopia. The city is administered by a city council and organized in 10 sub-cities and 117 districts. There are about 1303 banks found in Addis Ababa in 2016/2017 and an estimated number of more than 10,000 employees who worked in those banks [12].

The source of the study’s population was all bank workers engaged in bank activity in Addis Ababa, Ethiopia. The study population was all bank workers engaged in bank activity in the selected three sub-cities of Addis Ababa. For this study, bank workers who worked at least 1 year were included.

### Sample size and sampling procedures

The sample size for the magnitude of WMSDs was computed based on single proportion formula by using proportion of WMSDs from a previous study conducted in Rwanda among bank workers who accounted for 45.8% [13] and a 95% confidence interval (CI) and margin of error of 5% and 10% non-response rate. Since the sampling method was multi-stage, this study used design effect of 2 making a total sample size of 838.

For associated factors for the occurrence of WMSDs, the sample size was computed using double population proportion formula using Epi Info software considering the different variables like sitting while back is bent, sitting while back is twisted, and work time break. But the sample sizes were low compared to the sample size for the prevalence of WMSDs. Finally, the larger sample size was taken for this study.

Study participants were selected using multi-stage sampling techniques. From 10 sub-cities, three sub-cities (Arada, Nifas silk lafto, and Kirkos) was selected randomly using lottery method. Then, the number of banks was determined in each sub-city proportionally. By using probability proportional to size (PPS) study subjects were allocated to each bank and selected by a simple random sampling technique.

### Data collection tools

The standard Nordic Musculoskeletal Questionnaire (NMQ) was used to assess the magnitude of neck, shoulder, upper back, lower back, hip/thigh, knee/leg, ankle/foot, wrist/hand, and elbow musculoskeletal disorders [14]. The reliability of the Amharic version questionnaire was tested using Cronbach’s alpha (Cronbach’s alpha = 0.72). Detailed information regarding socio-demographic factors, behavioral factors, ergonomic factors, organizational, and psychosocial factors was also included in the structured questionnaire.

### Data quality control

The data collection tool was first designed in English and then translated in to the local language, Amharic and then back to English. Afterward, training was provided for four data collectors and two supervisors for 2 days, which made them familiar with the data collection procedures. Finally, a pre-test was done on 5% (*N* = 42) of the total sample size. This was done so that those who did not participate in the main study would be addressed. Based on the pre-test analysis, unclear questions were modified. Supervisors and investigators evaluated the collected data completeness, accuracy, and clarity on a daily basis.

### Data processing and analysis

The data entry and code was done by Epi Info version 7 and exported to SPSS version 20 for analysis. The outcome variable (WMSDs) coded as No = 0 and Yes = 1. Descriptive analysis was done for both dependent and independent variables and binary logistic regression model was used to see the statistical association between different predictors and outcome variables. In this study, 18 variables with *p* value < 0.2 in bivariate analysis were included in multivariable logistic regression analysis model to control the effect of confounders. Model fitting was checked by using Hosmer and Lemeshow goodness of fit test, which showed *p* value = 0.88. Finally, adjusted odds ratio (AOR) with 95% CI and *p* value < 0.05 was used to establish the significance of association between explanatory variables and dependent variable.

### Operational definitions

- *Bank workers*. Employees that perform financial activities that includes supervisor, customer service, public relation, accounting clerks, loan officers, and managers
- *Work*-*related musculoskeletal disorders*. Bank workers having perceived pain, ache. or discomfort for at least 2–3 workdays in the last week or the last 12 months in any part of their bodies segments was considered [9]
- *Body mass index* (*BMI*). < 18.5 = underweight, 18.5–24.9 = normal, 25.0–29.9 = overweight, and ≥ 30.0 = obese [15]
- *Perform physical activity*. Exercising or doing any kind of sport activity including walking at least 150 min/week [16, 17].
- *Awkward posture*. Bank workers perform activities with the neck bent more than 30 degrees without support, working with a bent wrist, working with the back bent without support, and squatting and kneeling for 2 or more hours continuously [18].
- *Repetitive work*. Perform work by repeating the same activity with less than 30 s or no variation every few seconds for 2 or more hours [18]
- *Fixed postures*. Bank workers perform activities by prolonged sitting in a limited space for 2 or more hours without changing positions [18].
- *Job satisfaction*. A score measured using the generic job satisfaction scale as yes (32–50) and no (10–31) [19]
- *Job stress*. A score measured using the workplace stress scales moderate to severe job stress as yes (16–40) and no (≤ 15) [20]

## Results

### Socio-demographic characteristics of the study participants

A total of 755 bank workers returned and fully answered questionnaires making a response rate of 90%. Of the total study participants, 50.7% (*N* = 383) were females. The mean age of the study participants with standard deviation (SD) were 29.4 (SD = 5.91) years. Majority of the study participants, 78.5% (*N* = 593) religion was orthodox and more than three fourths of them had a Bachelor’s degree, 75.4% (*N* = 569). More than half, 51.2% (*N* = 386), of the study participants were single in their marital status. About 64.6% (*N* = 488) of the participants’ job was customer service. The mean monthly income of the participants was 8794.4 (SD = 5544.0) Ethiopian Birr. About 40.5% (*N* = 305) of the respondents worked at the bank for over 10 years (Table 1).

**Table 1:** Socio-demographic characteristics of bank workers, Addis Ababa, Ethiopia, 2018 (*N* = 755)

Variables/characteristics	Numbers	Percent (%)
**Sex**		
Female	383	50.7
Male	372	49.3
**Age**		
20–29	477	63.2
30–39	235	31.1
≥ 40	43	5.7
**Religion**		
Orthodox	593	78.5
Muslim	54	7.2
Protestant	88	11.7
Catholic	20	2.6
**Educational status**		
Certificate	8	1.1
Diploma	60	8.0
Bachelor’s degree	569	75.4
Master	118	15.6
**Marital status**		
Single	386	51.2
Married	367	48.6
Separated	1	0.1
Divorced	1	0.1
**Job category**		
Accounting clerks	34	4.5
Customer service	488	64.6
Managers	108	14.3
Others	125	16.6
**Salary**		
≤ 5240	196	26.0
5241–8500	212	28.1
8501–11,200	177	23.5
> 11,200	169	22.4
**Work experience**		
1–5	162	21.5
6–9	287	38.1
≥ 10	305	40.5

### Magnitude of self-reported work-related musculoskeletal disorders among bank workers

**Fig. 1:**
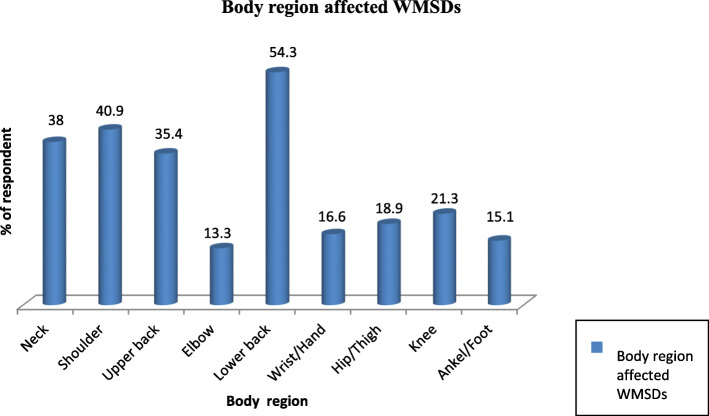
The frequency of self-reported body regions affected by WMSDs among bank workers, Addis Ababa, Ethiopia, 2018

The result of the study revealed that from the total amount of bank workers 77.6% [95% CI 75–81%] suffered work-related musculoskeletal disorders at least at one region of the body in the last 1 year. The four most effected body regions that the respondents reported pain or discomfort in or around were the lower back 54.3% (*N* = 410), shoulder 40.9% (*N* = 309), neck 38.0% (*N* = 287), and upper back 35.4% (*N* = 267), while the lowest effected body parts that the respondents reported were hip/thigh 18.9% (*N* = 143), wrist/hand 16.6% (*N* = 125), and ankle/foot 15.1% (*N* = 114) (Fig. 1).

### Behavioral characteristics of bank workers

The result indicated that the majority of bank workers 96.6% (*N* = 729) were non-smokers. Regarding alcohol use 19.7% (*N* = 149) of the respondents drank alcohol below the threshold of two times per week. The result also indicated that 45.8% (*N* = 346) of respondents did not practice physical activities. More than half of the participants, 69.0% (*N* = 521) had BMI in the normal range. A majority of respondents 94.3% (*N* = 712) used their right hand to perform work-related duties, and also 96% (*N* = 725) of bank workers did not show musculoskeletal disorder symptoms before they started bank work (Table 2).

**Table 2:** Behavioral characteristics of bank workers in Addis Ababa, April 2018 (*N* = 755)

Variables/characteristics	Numbers	Percent (%)
**Smoker**		
No	729	96.6
Yes	26	3.4
**Alcohol consumption**		
≥ Two times per week	47	6.2
< Two times per week	149	19.8
Never	559	74.0
**Physical activities**		
No	346	45.8
Yes	409	54.2
**BMI**		
Normal	521	69.0
Underweight	108	14.3
Overweight	103	13.7
Obese	23	3.1
**Dominant hand**		
Right hand	712	94.3
Left hand	43	5.7
**History of MSDs**		
No	725	96.0
Yes	30	4.0

### Working environment, ergonomic characteristics, and psychosocial factor of bank workers

The study result showed that 92.8% (*N* = 701) of bank workers performed activities at least 6 days per week. Majority, 97% (*N* = 732), of bank workers performed their work habits by sitting more than or equal to 2 h/day. In addition, majority 40.8% (*N* = 308) of bank workers were sitting with their back(s) bent. The result also indicated that 94.7% (*N* = 715) of bank workers performed work by using a computer. More than half (52.1%) (*N* = 393) of the workers performed activities with a fixed position. Moreover, most bank workers, 87.3% (*N* = 659), used movable chairs and 77.9% (*N* = 588) of bank workers used arm rest chair. The finding also indicates that most bank workers, 95.1% (*N* = 718) did not take work break in minutes. More than half, 59.7 % (*N* = 451), did repetitive activities less than 30 s and only 10.7% (*N* = 81) of the bank workers took ergonomic training. About 87.0% (*N* = 657) of the bank workers had a good relationship with their customers. Furthermore, 71.3% (*N* = 538) of respondents had a good relationship with their boss (Table 3).

**Table 3:** Working environment, ergonomic characteristics, and psychosocial factors of bank workers in Addis Ababa, April 2018 (*n* = 755)

Variables/characteristics	Number	Percent (%)
**Working days**		
≤ 6 days	701	92.8
7 days	54	7.2
**Time spent sitting position**		
< 2 h	23	3
≥ 2 h	732	97
**Type of sitting posture**		
Back twisted	205	27.2
Back bent	308	40.8
Back straight	242	32
**Utilization of computer**		
No	40	5.3
Yes	715	94.7
**Fixed position**		
No	362	47.9
Yes	39	52.1
**Type of chair**		
Fixed chair	96	12.7
Movable chair	659	87.3
**Arm rest chair**		
No	167	22.1
Yes	588	77.9
**Work break in min**		
No	718	95.1
Yes	37	4.9
**Repetitive activities less than 30 s**		
No	304	40.3
Yes	451	59.7
**Ergonomic trainning**		
Yes	81	10.7
No	674	89.3
**Customer relationship**		
Good	657	87.0
Poor	98	13
**Boss relationship**		
Good	538	71.3
Fair	165	21.9
Poor	24	3.2
None	28	3.7
**Colleague relationship**		
Good	629	83.3
Fair	114	15.1
Poor	12	1.6
**Job stress**		
No	72	9.5
Yes	683	90.5
**Job satisfaction**		
No	326	43.2
Yes	429	56.8

### Factors associated with self-reported work-related musculoskeletal disorders

A binary logistic regression analysis was employed to assess the effect of explanatory variables to dependent variables and 18 variables that had *p* value less than 0.2 were included in the multivariate analysis.

Study participants who were female were 3 times more likely to develop WMSDs than male participants [AOR = 2.98, 95% CI 1.91–4.65]. Respondents sitting with their back twisted were 3.6 times more likely to develop WMSDs [AOR = 3.59, 95% CI 2.13–6.08], and respondents sitting with their back bent were 4 times more likely to develop WMSDs [AOR = 4.06, 95% CI 2.48–6.66] when compared to sitting back straight. Moreover, respondents who worked with fixed position were 1.8 times more likely to develop WMSDs [AOR = 1.78, 95% CI, 1.17–2.71] than those who worked without fixed position. Participants sitting in fixed chairs were 2.6 times more likely to develop WMSDs [AOR = 2.62, 95% CI 1.19–5.75] than those who sat in movable chairs. And respondents who had no work time breaks were 3 times more likely to develop WMSDs [AOR = 3.33, 95% CI 1.44–7.71] than those who had work time breaks. Furthermore, respondents who had job stress were 2 times more likely to develop WMSDs [AOR = 2.33, 95% CI 1.19–4.54] than workers who had no job stress (Table 4).

**Table 4:** Multi variable analysis of factors associated with work-related musculoskeletal disorders among bank workers, Addis Ababa, Ethiopia, 2018 (*N* = 755)

Variable	Frequency		COR (95% CI)	AOR (95% CI)
	No	Yes		
**Sex**				
Female	51	332	3.02 [2.09–4.36]	2.98 [1.91–4.65]**
Male	118	254	1.00	1.00
**Age**				
20–29	115	362	1.00	1.00
30–39	49	186	1.21 [0.83–1.76]	1.09 [0.66–1.83]
≥ 40	5	38	2.414 [0.92–6.27]	3.05 [0.86–10.73]
**Educational status**				
Certificate	4	4	0.17 [0.04–0.74]	0.260 [0.03–1.821]
Diploma	21	39	0.31 [0.14–0.65]	0.949 [0.30–2.98]
Bachelor’s degree	127	442	0.58 [0.33–1.01]	0.666 [0.33–1.32]
Master	17	101	1.00	1.00
**Job catagory**				
Accounting clerks	14	20	0.32 [0.14–0.73]	0.23 [0.06–0.79]
Customer service	115	373	0.73 [0.44–1.20]	0.85 [0.46–1.57]
Managers	17	91	1.20 [0.60–2.40]	1.56 [0.65–3.73]
Others	23	102	1.00	1.00
**Salary**				
≤ 5240	58	139	0.47 [0.28–0.79]	0.93 [0.46–1.90]
5241–8500	48	164	0.67 [0.40–1.13]	0.92 [0.47–1.78]
8501–11200	35	142	0.80 [0.46–1.39]	1.04 [0.54–2.01]
> 11200	28	141	1.00	1.00
**Smoking behavior**				
No	166	563	1.00	1.00
Yes	3	23	2.26 [0.67–7.62]	2.57 [0.47–14.08]
**BMI**				
Normal	117	404	1.00	1.00
Underweight	20	83	1.27 [0.75–2.15]	1.01 [0.54–1.89]
Overweight	30	73	0.70 [0.44–1.13]	0.82 [0.46–1.48]
Obese	2	21	3.04 [0.70–13.15]	3.10 [0.60–15.87]
**History of MSDs**				
No	167	558	1.00	1.00
Yes	2	28	4.19 [0.98–17.77]	3.46 [0.68–17.48]
**Type of sitting position**				
Back twisted	28	177	4.76 [2.96–7.65]	3.59 [2.13–6.08]**
Back bent	37	271	5.52 [3.60–8.46]	4.06 [2.48–6.66] **
Back straight	104	138	1.00	1.00
**Utilization of computer**				
No	15	25	1.00	1.00
Yes	154	561	2.18 [1.12–4.24]	2.38 [0.94–5.99]
**Work break in min**				
No	148	570	5.05 [2.57–9.93]	3.33 [1.44–7.71]**
Yes	21	16	1.00	1.00
**Repetitive motion**				
No	83	221	1.00	1.00
Yes	86	365	1.59 [1.12–2.25]	1.15 [0.74–1.78]
**Fixed position**				
No	107	255	1.00	1.00
Yes	62	331	2.24 [1.57–3.18]	1.78 [1.17–2.71]**
**Boss relation**				
Good	138	400	1.00	1.00
Fair	21	144	2.36 [1.44–3.89]	1.97 [1.00–3.56]
Poor	3	21	2.41 [0.71–8.22]	1.18 [0.31–4.45]
None	7	21	1.03 [0.43–2.49]	1.90 [0.64–5.64]
**Customer relation**				
Good	159	498	1.00	1.00
Poor	10	88	2.81 [1.42–5.53]	1.91 [0.86–4.25]
**Type of chairs**				
Fixed	11	85	2.44 [1.26–4.68]	2.6 [1.19–5.75]**
Movable	158	501	1.00	1.00
**Job satisfaction**				
No	60	266	1.51 [1.06–2.15]	0.98 [0.62–1.53]
Yes	109	320	1.00	1.00
**Job stress**				
No	30	42	1.00	1.00
Yes	139	544	2.79 [1.69–4.63]	2.33 [1.19–4.54]*

*Others* public relations, loan officers, supervisors; *AOR* adjusted odd ratio; *CI* confidence interval

*Statistically significant at *p* value < 0.05

**Statistically significant at *p* value < 0.001

## Discussion

Findings of this study reveal that 77.6% [95% CI 75–81%] of bank workers suffered work-related musculoskeletal disorders. This study was consistent with a study conducted among bank workers in Kuwait (80%) [9]. But higher compared to the studies done in Nigeria 71.6% [10], India 37% [21], and Thailand 63% [22].

By contrast, the current study had lower magnitude as compared to the study conducted in India/Punjab 83.5% [8] and Ghana 83.5% [11]. The difference might be due to difference in sociocultural factors, job stress [21], assessment tools, workload, ergonomic design of work station, and sedentary activity of participants [11].

The result of this study revaled that female respondents were 3 times more likely to develop WMSDs than male participants. This was similar to a study conducted in Kuwait [9], India [21], Ghana [11], Sri Lanka [23], and Nigeria [10]. However, this study was inconsistent to a study conducted among bank workers in India [8] and Bangladesh [24]. This disparity might be due to work load and sociocultural factor [21, 25].

In this study, it has been observed that the type of sitting position was an important predictor of WMSDs among bank workers. Respondents sitting with their back twisted were 3.6 times more likely to develop WMSDs than sitting with their back straight. This finding was consistent with studies done in Rwanda [13] and Dutch [26]. On the other hand, respondents sitting with their back bent were 4 times more likely to develop WMSDs than sitting with a straight aligned back. This finding was in agreement with studies done in Rwanda [13], Sri Lanka [23], Dutch [26], and China [27]. This is due to the fact that poor posture can bring stiffness and compression over all muscle and skeletal areas causing aching and discomfort of body regions [28, 29].

In addition to this, respondents who worked with a fixed position were 1.8 times more likely to develop WMSDs than those workers who worked without fixed position. This was consistent with different studies done on WMSDs of the neck, lower back, and shoulder regions of the body [10, 23, 30–33]. This might because when workers work in a fixed position, the muscle has no opportunity to relax and it restricts the flow of blood [34]

Respondents who had no work time breaks were 3 times more likely to develop WMSDs than those who had work time break. The findings of this study were consistant with the study conducted in Ethiopia [35], Rwanda [13], and China [27]. Most of the participants of this study used movable chairs to perform their work time duties, although sitting in fixed chairs had 2.6 times more likely to develop WMSDs than sitting on movable ones.

Furthermore, respondents who had job stress were 2 times more likely to develop WMSDs than workers who had no job stress. This finding was consistent with studies done among bank workers in Kuwait [9]. This could be due to the fact that high stress may increase muscle tension and decrease micro pauses in muscle activity [11].

Though the study did its best to indicate the magnitude of WMSDs among bank workers, it is not free from limitations. The cross-sectional design might have prevented the work from showing temporal relationships. In addition, since WMSDs has not been verified by clinical diagnosis in the last 12 months, our result is based on self-reporting. Thus, it is possible that participants failed to remember correctly and ultimately end up in a recall bias. In addition, this study did not assess the amount of personal computer and smart phone usage in private and business.

## Conclusion

The finding of this study has shown that bank workers highly suffered from work-related musculoskeletal disorders at least in one region of the body in the previous 12 months. Of these, high prevalent body regions were the lower back followed by the shoulder, neck, and upper back. The multivariate analysis indicated that the significant predictors for the occurrence of WMSDs among bank workers were being female, awkward posture, static position, no work time break, type of chairs and job stress. In order to reduce the problem, bank workers should employ proper types of chairs, practice proper work posture, healthy working conditions, and create awareness programs on how to maintain healthy conditioning and to have enough leisure time.

## Supplementary information

**Additional file 1: Table 1.** Socio-demographic characteristics of bank workers, Addis Ababa, Ethiopia, 2018 (N = 42). **Table 2.** Behavioral characteristics of bank workers in Addis Ababa, April 2018 (n=42).

## Data Availability

All data generated or analyzed during this study are included in this article. The data that support the findings of this study are also available from the corresponding authors upon reasonable request.

## Abbreviations

AOR: Adjusted odd ratio
CI: Confidence interval
BMI: Body mass index
ILO: International Labor Organization
NMQ: Nordic Musculoskeletal Questionnaire
OSHA: Occupational Safety and Health Administration
SD: Standard deviation
SPSS: Statistical Package for Social Science
WMSDs: Work-related musculoskeletal disorders
WHO: World Health Organization
