# Supplementary material for: Work-related musculoskeletal disorders and associated factors among bank workers in Addis Ababa, Ethiopia: a cross-sectional study
Source: Environ Health Prev Med. 2020 Jul 27;25:33. doi: 10.1186/s12199-020-00866-5 (PMC7385884; doi:10.1186/s12199-020-00866-5)
Supplement: Supplementary file 1 — Additional file 1: Table 1. Socio-demographic characteristics of bank workers, Addis Ababa, Ethiopia, 2018 (N = 42). Table 2. Behavioral characteristics of bank workers in Addis Ababa, April 2018 (n=42). [file 12199_2020_866_MOESM1_ESM.docx]

**Work Related Musculoskeletal Disorders and Associated Factors among Bank Workers in Addis Ababa Ethiopia: A Cross Sectional Study**

**Pre test result**

**Table 1:** Socio-demographic characteristics of bank workers, Addis Ababa, Ethiopia, 2018 (N = 42)

| **Variables/ characteristics** | **Numbers** | **Percent (%)** |
| --- | --- | --- |
| **Sex** |  |  |
| Female | 15 | 35.7 |
| Male | 27 | 64.3 |
| **Age** |  |  |
| 20-29 | 25 | 59.5 |
| 30-39 | 15 | 35.5 |
| ≥40 | 2 | 4.8 |
| **Religion** |  |  |
| Orthodox Muslim Protestant  Catholic | 35  3  1  3 | 83.3  7.1  2.4  7.1 |
| **Educational Status** |  |  |
| Certificate  Diploma  Bachelor’s degree  Master | 0  2  32  2 | 0  4.8  76.2  19 |
| **Marital Status** |  |  |
| Single | 18 | 42.9 |
| Married | 24 | 57.1 |
| Separated | 0 | 0 |
| Divorced | 0 | 0 |
| **Job category** |  |  |
| Accounting clerks | 1 | 2.4 |
| Customer service | 27 | 64.3 |
| Managers | 5 | 11.9 |
| Others | 9 | 21.4 |
| **Salary** |  |  |
| ≤5240 | 6 | 14.3 |
| 5241-8500 | 21 | 50.0 |
| 8501-11200 | 8 | 19.0 |
| >11200 | 7 | 16.7 |
| **Work experience** |  |  |
| 1-5 | 8 | 19 |
| 6-9 | 19 | 45.2 |
| ≥10 | 15 | 35.7 |

**Table 2**: Behavioral characteristics of bank workers in Addis Ababa, April 2018 (n=42)

| **Variables/ characteristics** | **Numbers** | **Percent (%)** |
| --- | --- | --- |
| **Smoker** | | |
| No  Yes | 40  2 | 95.2  4.8 |
| **Alcohol consumption** |  |  |
| ≥ Two times per week  <Two times per week  Never | 9  11  22 | 21.4  26.3  52.3 |
| **Physical activities** |  |  |
| No  Yes | 22  20 | 52.3  47.7 |
| **BMI** |  |  |
| Normal  Underweight  Overweight  Obese | 31  5  4  2 | 73.8  11.9  9.5  4.8 |
| **Dominant hand**  Right hand  Left hand | 40  2 | 95.2  4.8 |
| **History of MSDs**  No  Yes | 39  3 | 92.9  7.1 |
